# Single-cell analysis of fetal testis reveals dysfunction of human Leydig cells in Klinefelter syndrome

**DOI:** 10.1172/JCI201124

**Published:** 2026-06-09

**Authors:** Tong Yan, Guancheng Chen, Jie Zhang, Wenjing Jia, Nan Lu, Shuping Jin, Haotian Zhang, Yichen Zhao, Lu Jiang, Jing Wu, Qing Liu, Chenghao Situ, Hui Zhu, Yan Li, Quan Wang, Xiaoyu Yang, Chao Qin, Xiaofeng Song, Qing Cheng, Xuejiang Guo

**Affiliations:** 1 Department of Histology and Embryology, State Key Laboratory of Reproductive Medicine and Offspring Health, Nanjing Medical University, Nanjing, Jiangsu, China.; 2 Department of Gynecology, The First Affiliated Hospital of Nanjing Medical University, Nanjing, Jiangsu, China.; 3 Department of Biomedical Engineering, Nanjing University of Aeronautics and Astronautics, Nanjing, Jiangsu, China.; 4 State Key Laboratory of Reproductive Medicine and Offspring Health, Clinical Center of Reproductive Medicine, First Affiliated Hospital, Nanjing Medical University, Nanjing, Jiangsu, China.; 5 Department of Obstetrics and Gynecology, Women’s Hospital of Nanjing Medical University, Nanjing Women and Children’s Healthcare Hospital, Nanjing, Jiangsu, China.; 6 School of Biomedical Engineering and Informatics and; 7 Department of Clinical Laboratory, Sir Run Run Hospital, Nanjing Medical University, Nanjing, Jiangsu, China.; 8 State Key Laboratory of Reproductive Medicine and Offspring Health, Department of Urology, The First Affiliated Hospital of Nanjing Medical University, Nanjing, Jiangsu, China.

**Keywords:** Cell biology, Reproductive biology, Embryonic development

## Abstract

Klinefelter syndrome (KS), the most common sex chromosome aneuploidy (affecting approximately 1 in 650 live male births), causes severe infertility. The extra X chromosome can impair the development of fetal germ cells, but its effects on somatic cells, especially Leydig cells, are still not well known. We performed single-cell RNA-sequencing analysis of fetal KS and control testicular cells and found 2 clusters of KS Sertoli cells, with the *XIST*-negative cluster showing distinct gene expression pattern and abnormally increased G2/M ratio. Fetal KS Leydig cells showed increased proliferation and immature differentiation with high level of MAPK signaling pathway and X-linked *EIF1AX*. Inhibition of MAPK signaling partially rescued overproliferation and defective differentiation and androgen secretion in KS Leydig cells, while overexpression of *EIF1AX* recapitulated the phenotypes of increased proliferation and decline in testosterone synthesis capacity in the Leydig cell line. These findings reveal the early pathological mechanisms of KS somatic cells and lay the groundwork for developing early intervention strategies.

## Abstract 2

MAPK pathway and EIF1AX are upregulated in Leydig cells of fetal testis with Klinefelter syndrome, leading to excessive proliferation and impaired differentiation.

## Introduction

Chromosomal abnormalities account for more than 80% of the genetic etiologies of birth defects (1–3). A common sex chromosome aneuploidy with congenital testicular hypoplasia, Klinefelter syndrome (KS) has an incidence of 1 in 650 newborn males (4). Its typical manifestations include tall stature, hypogonadism, gynecomastia, azoospermia, and metabolic abnormalities. Due to the difficulty in early detection during childhood and adolescence, studies of KS are mainly focused on the adult stage (5, 6). Early hypotheses suggested that aneuploid germ cells at meiosis fail to pair correctly, leading to apoptosis (7), but it has also been found that aneuploid cells can sometimes mature into spermatozoa (8). KS shows impaired spermatogenesis and immature Sertoli and Leydig cells (9, 10). Loss of the extra X chromosome is important for the maturation of Sertoli cells and focal spermatogenesis (11–13). Some genes were found to be involved in sex chromosome dosage compensation and somatic cell maturation. The transcriptome and methylome analysis of KS blood showed epigenetic changes and upregulation of 11 X-linked genes, including *EIF1AX* (14). The studies of KS are mainly focused on the abnormal development of spermatogenic cells. Studies of the defects of somatic cell microenvironment are still limited.

In fetal stage, testicular degeneration in KS begins in midgestation, mainly characterized by a significant reduction in the number of germ cells (15, 16), especially MAGEA4-positive prespermatogonia (17). Recently, Lu et al. found heightened pluripotency and reduced differentiation potential of fetal germ cells in KS, attenuated interactions between Sertoli cells and germ cells, and impaired migration of fetal germ cells and Sertoli cells to the base of the testicular cord (18). However, the defective regulation of KS somatic cells, including Sertoli cells in close contact with the germ cells and Leydig cells responsible for testosterone production, is still not well characterized in the fetal stage.

Here, we performed single-cell RNA-sequencing (scRNA-seq) analysis of fetal and adult testicular tissues from individuals with KS and identified abnormalities in Leydig cell proliferation and testosterone synthesis capacity. We found that the X-linked *EIF1AX* is abnormally upregulated in Leydig cells, and its overexpression leads to abnormal proliferation and testosterone synthesis in Leydig cells. Using ex vivo cultures of fetal KS testicular tissue, inhibition of MAPK signaling pathway can partially rescue the defective Leydig cell proliferation and testosterone synthesis. Leydig cell dysfunction and developmental abnormalities are important pathological contributors to fertility disorders in KS.

## Results

### Single-cell transcriptome analysis of fetal testis with KS.

**Figure 1:**
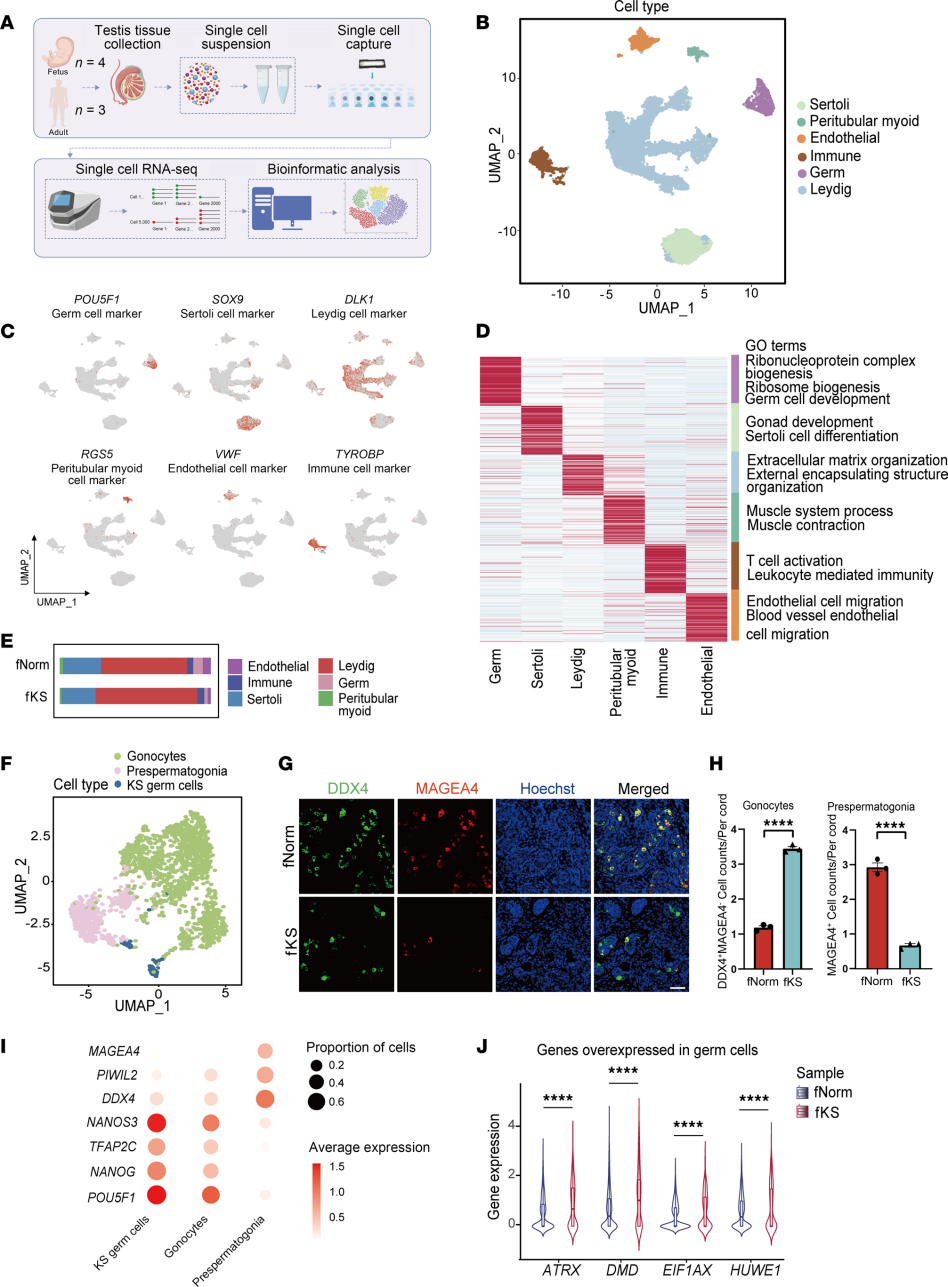
Single-cell transcriptome profiling and analysis of KS and loss of prespermatogonia in KS testis. (**A**) Schematic diagram of experimental workflow. Fetal samples (KS *n* = 2, control *n* = 2) and adult samples (KS *n* = 2, control *n* = 1). (**B**) Uniform manifold approximation and projection (UMAP) plot illustrating the major cell types (*n* = 38,952 cells) from KS samples (*n* = 2) and samples from aborted male fetuses (*n* = 2). Each dot corresponds to an individual cell, and the colors denote different cell types. (**C**) Expression patterns of marker genes displayed on the UMAP plot. (**D**) Heatmap of marker genes in each cluster and GO term enrichment. (**E**) Fraction of each cell types isolated from KS and normal samples. (**F**) UMAP plot of all germ cells from the embryonic sample (*n* = 1,866 cells, 4 samples). Each dot corresponds to an individual cell, and the colors denote different cell types. (**G**) Immunofluorescence costaining of DDX4 (green) and MAGEA4 (red). Scale bars, 50 μm. (**H**) The number of DDX4^+^MAGEA4^–^ cells and MAGEA4^+^ cells in the fetal gonads of male control fetuses and KS fetuses. Data are mean ± SEM. Statistical analysis was performed using unpaired 2-sided *t* tests. *n* = 15 regions from 3 independent samples or 20 regions from 3 independent samples in each group. (**I**) Dot plot showing the expression of selected gene markers for different stages of germ cells. (**J**) The expression level of 4 selected genes located on the X chromosome with significant difference between control and KS samples. Statistical analysis was performed using 2-sided Wilcoxon’s rank-sum test; ****P < 0.0001.

We isolated single cells from 4 fetal testis samples at 20–21 weeks postfertilization (Supplemental Table 1; supplemental material available online with this article; https://doi.org/10.1172/JCI201124DS1) and performed scRNA-seq using microfluidic chip–based microwell technology (19) (Figure 1A and Supplemental Figure 1A). After standard quality control, we obtained a total of 38,952 high-quality cells retained for downstream analysis, with 1,501–32,391 reads/cell and 670–5,988 genes/cell (Supplemental Figure 1, B and D). When all scRNA-seq cells were projected into low-dimensional subspaces, we found 20 clusters and annotated them into 6 cell types (Figure 1B). Based on marker genes, 6 major cell types were designated as Leydig cells (*DLK1*), Sertoli cells (*SOX9*), germ cells (*POU5F1*), endothelial cells (*VWF*), immune cells (*TYROBP*), and peritubular myoid cells (*RGS5*) (Figure 1C and Supplemental Figure 1F). To validate the accuracy of our cell type annotation, we performed the enrichment analysis of Gene Ontology (GO) terms on these clusters (Figure 1D and Supplemental Table 2). These enrichment results were consistent with the cell types annotated in marker genes.

To characterize major cell type differences between fetal KS and normal samples, we further analyzed their cell type proportions. Notably, the ratio of primordial germ cells was reduced in KS fetal testis, while Leydig cells exhibited a higher proportion than normal (Figure 1E and Supplemental Figure 1G). To reinforce the robustness of our findings, we performed H&E and immunofluorescence (IF) staining of fetal KS and control testes and observed a consistently significant decrease in the number of DDX4-positive cells (indicative of germ cells) in the KS group (Supplemental Figure 1E). Primordial germ cells differentiate into gonocytes in the first trimester of pregnancy and further differentiate into MAGEA4-positive prespermatogonia in the second and third trimesters of pregnancy (20, 21). According to cell markers, we observed 2 types of germ cells, gonocytes (*POU5F1*) and prespermatogonia (*MAGEA4*), in the control and KS testis (Figure 1, F and I, and Supplemental Figure 2, A and B). To confirm the development change of KS germ cells, we performed IF analysis, and we observed a significantly decreased number of MAGEA4-positive prespermatogonia and increased number of MAGEA4-negative germ cells in the KS testis (Figure 1, G and H), consistent with Lu et al.’s findings of differentiation abnormalities of fetal germ cells (17, 18). These results suggest that the differentiation potential of germ cells in KS fetal testis is severely impaired.

We assessed the gene expression changes between KS and control samples by analyzing differentially expressed genes (DEGs). We found 1,125 DEGs in germ cells (Supplemental Figure 2C). GO enrichment analysis showed enrichment of gonadal development and endoplasmic reticulum processes in upregulated genes, and translation-related and apoptosis-related processes in downregulated genes, suggesting a general dysfunction of the fetal germ cells in KS (Supplemental Figure 2, D and E). One-eighth (78/609) of the upregulated genes in the KS germ cells, including *ATRX*, *DMD*, *EIF1AX*, and *HUWE1*, was located on the X chromosome. This finding suggests that the extra X chromosome leads to upregulation of X-linked genes, which may be associated with germ cell development abnormality (Figure 1J).

### Sertoli cell heterogeneity and defects in fetal KS testis.

**Figure 2:**
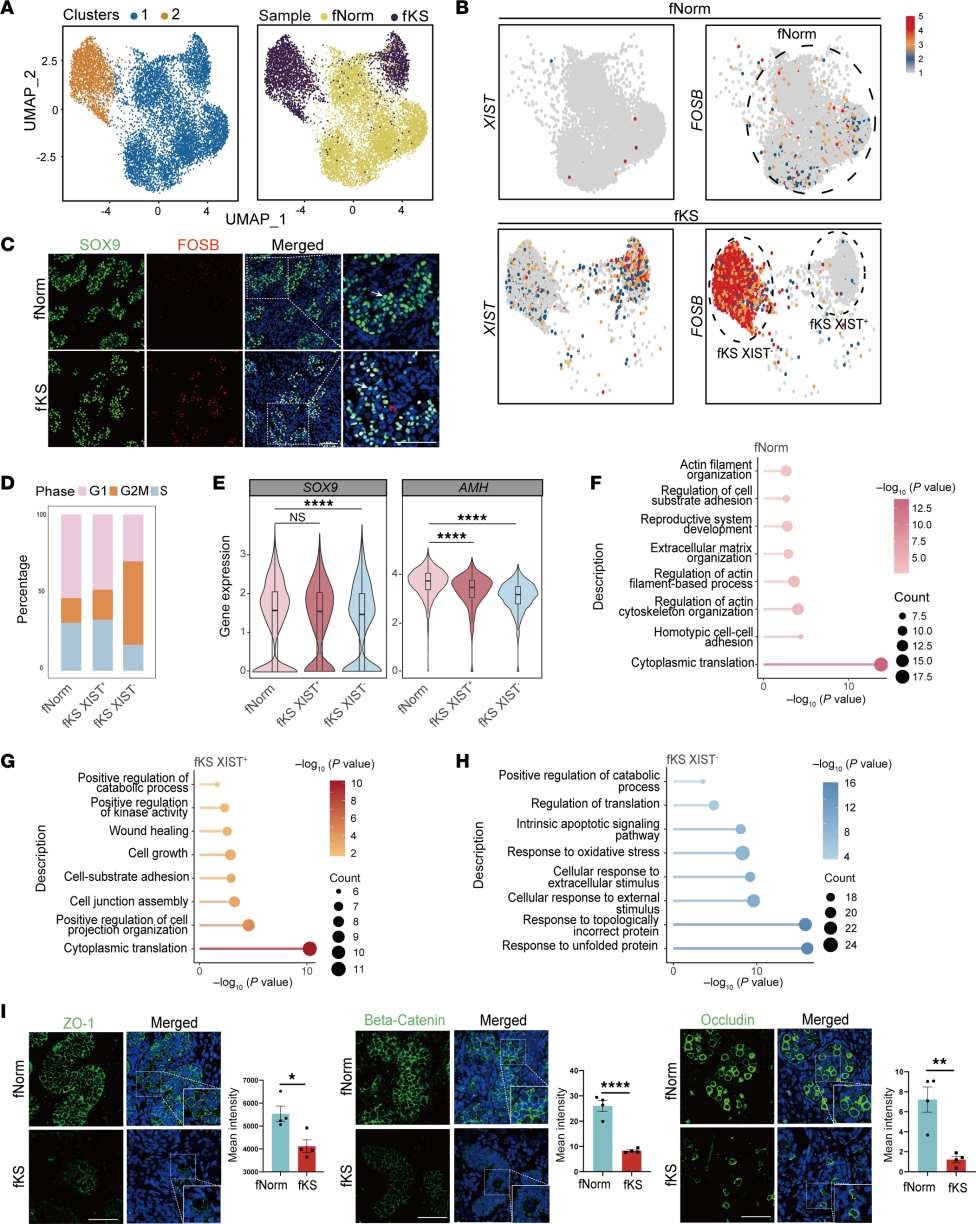
Defect and heterogeneity of fetal Sertoli cells in KS. (**A**) UMAP plot of all Sertoli cells (4 samples). Each dot corresponds to an individual cell, and colors represent different clusters and samples. (**B**) *XIST* and *FOSB* expression patterns drawn in UMAP. (**C**) Immunofluorescence costaining of SOX9 (green) and FOSB (red). Scale bars, 50 μm. White arrow indicates FOSB^–^ Sertoli cell, and red arrow indicates FOSB^+^ Sertoli cell. (**D**) Ratios of cells in G0, S, and G2/M phases. (**E**) The expression levels of *SOX9* and *AMH* were significantly different among control, *XIST*-positive, and *XIST*-negative KS samples. Statistical analysis was performed using 2-sided Wilcoxon’s rank-sum test; ****P < 0.0001. (**F**) GO terms enriched in fNorm Sertoli cells. The *x* axis is –log10(*P* value), and the size of the dots represents count. (**G**) GO terms enriched in fKS XIST^+^ Sertoli cells. The *x* axis is –log10(*P* value), and the size of the dots represents count. (**H**) GO terms enriched in fKS XIST^–^ Sertoli cells. The *x* axis is –log10(*P* value), and the size of the dots represents count. (**I**) Immunofluorescence staining of ZO-1, β-catenin, and occludin. Scale bars, 50 μm. Insets original magnification, ×600. Fluorescence signal intensities of 3 proteins are shown as mean ± SEM. Statistical analysis was performed using unpaired 2-sided *t* tests (*n* = 4).

It was reported that inactivation of the extra X chromosome is necessary for Sertoli cell maturation and focal spermatogenesis in adult testis (10), but the effect of the extra X chromosome on KS fetal Sertoli cells is still not well known. To evaluate the effects of the extra X chromosome on Sertoli cells at the fetal stage, we characterized the heterogeneity of the Sertoli cell subset by clustering, and we observed 2 clusters of Sertoli cells (Figure 2A). Specifically, cluster 1 contained both KS and normal Sertoli cells. Normal Sertoli cells with XY chromosomes were negative for *XIST*, while KS Sertoli cells with an extra X chromosome were positive for *XIST*, indicating important roles of suppression of the extra X chromosome by *XIST*. Cluster 2 was composed of only KS Sertoli cells negative for *XIST*. It seems that *XIST* activation is important for the normal development of KS Sertoli cells. In addition, we found that the expression of AP-1 complex members, such as *FOSB*, increased in XIST-negative cells (Figure 2B). We performed IF and observed both FOSB-positive and FOSB-negative Sertoli cells (Figure 2C), consistent with the 2 clusters of KS Sertoli cells identified by UMAP analysis. XIST-negative KS Sertoli cells were different from the normal Sertoli cells and showed induced expression of *FOSB*. FOSB stimulates S phase entry and induces cyclin D1 expression (22). We observed an abnormal increase in the proportion of G2/M-phase cells in *XIST*-negative KS Sertoli cells by cell cycle analysis, indicating abnormal cell proliferation (Figure 2D).

To elucidate the regulatory role of *XIST* expression on KS Sertoli cell function, we analyzed scRNA-seq data using 3 types of Sertoli cells. The expression of *SOX9* and *AMH* was significantly reduced in *XIST*-negative KS Sertoli cells (Figure 2E). GO enrichment analysis revealed that both control and *XIST*-positive KS Sertoli cells were enriched in pathways related to cytoskeletal and cell adhesion, suggesting that they shared similar molecular characteristics and retained key functions of normal Sertoli cells (Figure 2, F and G). In contrast, *XIST*-negative KS Sertoli cells were enriched in unfolded protein reactions, misfolded protein reactions, and apoptosis, indicating that these cells were more severely damaged (Figure 2H and Supplemental Table 3). β-Catenin is a marker for cell adherens junctions, while occludin and ZO-1 are markers for tight junctions (23–26). During the fetal period, the expression of certain junctional proteins can already be detected in the testis, and focal tight junctions begin to form between Sertoli cells (27, 28). To evaluate the function of KS Sertoli cells, we examined the expression of these 3 proteins by IF. All 3 proteins showed reduced expression in KS testis (Figure 2I). Together, our study reveals that *XIST* expression plays an important role in the KS Sertoli cell development defect.

### Abnormal intercellular communication network in fetal KS testis.

**Figure 3:**
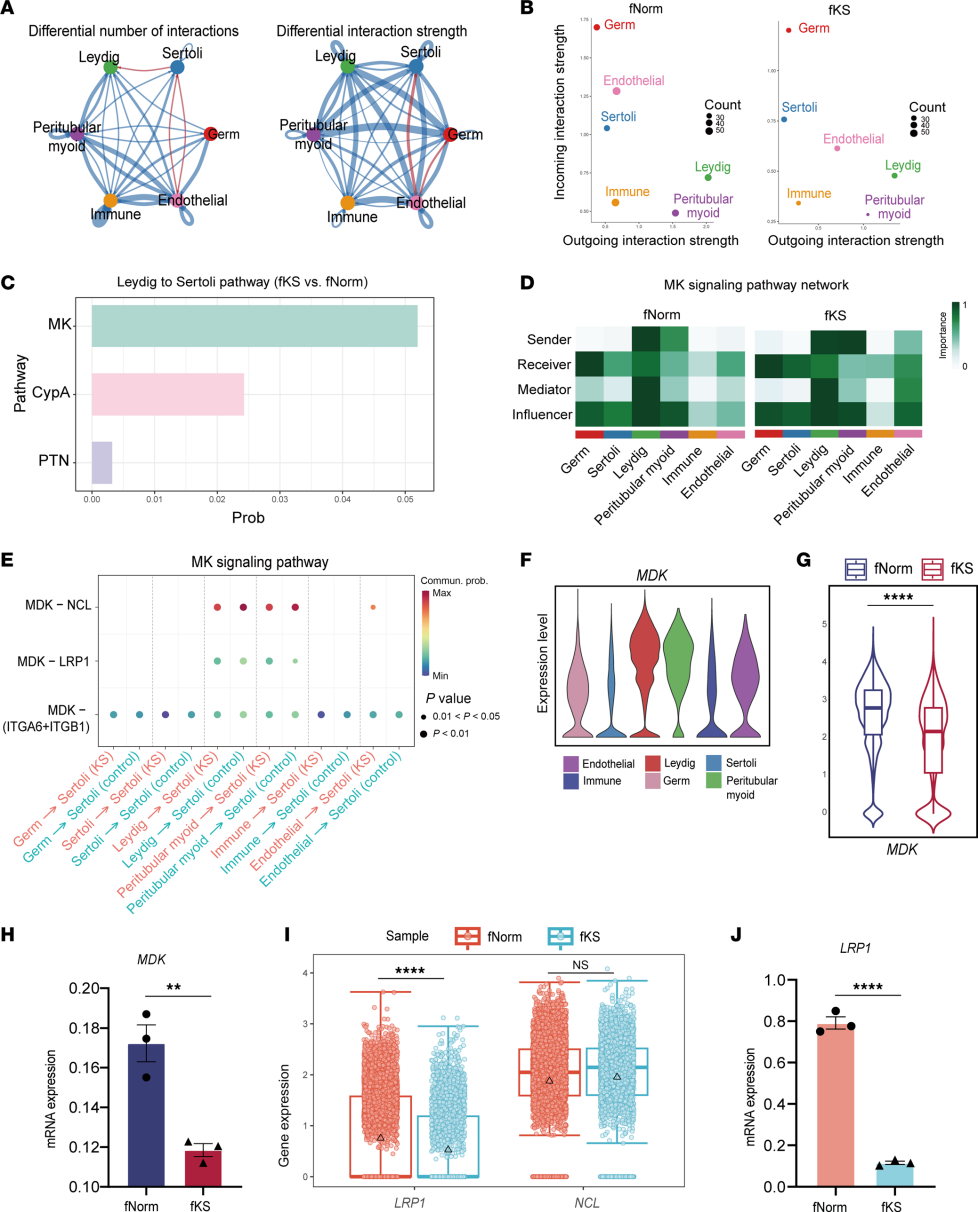
Intercellular interactions between Sertoli and other cell types in KS. (**A**) The circle diagrams depicting the relative number (left) or strength (right) of interactions in the cell-cell communication network between KS and control group. Red or blue edges represent an increase or decrease trend in signaling in KS group compared with control group, respectively. (**B**) Dot plot showing the relative strength of signaling pathways sent or received by each cell type (Endothelial, Sertoli, Peritubular myoid, Immune, Germ, Leydig). (**C**) Signaling pathways with the greatest variation between Sertoli cells and Leydig cells. PTN, pleiotrophin. (**D**) Heatmap shows the roles of different cell types in the MK signaling pathway. (**E**) Dot plot of ligand-receptor interactions in the MK pathway from different cell types to Sertoli in KS and control fetal testes. Commun. Prob., communication probability. (**F**) Expression of ligand *MDK* in testicular cell populations. (**G**) The expression of *MDK* in Leydig cells of KS and control. Statistical analysis was performed using 2-sided Wilcoxon’s rank-sum test; ****P < 0.0001. (**H**) The expression of *MDK* in control and KS primary Leydig cells using qRT-PCR. Data are presented as the mean ± SEM from 3 independent experiments. **P < 0.01. *GAPDH* was used as a loading control (*n* = 3). (**I**) The expression of *LRP1* and *NCL* in Sertoli cells of KS and control. Statistical analysis was performed using 2-sided Wilcoxon’s rank-sum test; ****P < 0.0001. The triangle (▲) represents the arithmetic mean in each group. (**J**) The expression of *LRP1* in control and KS primary Sertoli cells using qRT-PCR. Data are presented as the mean ± SEM from 3 independent experiments. ****P < 0.0001. *GAPDH* was used as a loading control (*n* = 3).

To evaluate which cells might affect the Sertoli cells, we analyzed the interactions among different cell populations using CellChat (29). The results showed that both the number and strength of interactions among testicular cell populations were generally reduced in KS testis (Figure 3A). The cell-cell communication patterns identified Leydig cells as the primary senders both in KS and in control samples (Figure 3B). Given the close anatomical proximity between Leydig cells and Sertoli cells, and that androgens secreted by Leydig cells regulate Sertoli cell functions via their androgen receptors to support spermatogenesis, we further analyzed the intercellular communication between these 2 cell types and found that the midkine (MK) pathway exhibited the most marked alteration in KS testis (Figure 3C and Supplemental Figure 2, F and G). Notably, our cell-cell communication analysis identified the MK and PTN signaling pathways as the most differentially activated pathways in KS, which strongly replicates the prior findings by Mahyari et al. (30), where MK and PTN signaling were also ranked as the top upregulated pathways in adult KS testis. Although the intensity of the major sender of MK pathway signals did not differ between control and KS Leydig cells, the intensity of signals received by Sertoli cells as receivers changed (Figure 3D). Detailed ligand-receptor pair analysis of the MK pathway showed attenuated MDK-NCL, MDK-LRP, and MDK-(ITGA6+ITGB1) (Figure 3E). Among these interactions, MDK-NCL and MDK-LRP pairs were mainly restricted to the Leydig, Sertoli, and peritubular myoid cells. MDK, the ligand of the MK signaling pathway, was predominantly expressed in control Leydig cells (Figure 3F) but was significantly reduced in KS Leydig cells (Figure 3G). We performed qRT-PCR quantification and observed consistent lower expression in KS Leydig cells (Figure 3H). Studies have shown that MDK could activate prosurvival signaling through LRP receptor internalization and nucleolin-dependent translocation to the nucleus (31, 32). The MK signaling pathway has been also observed in species such as sheep, buffalo, and mice (33, 34). As for receptors of the MK signaling pathway, *LRP1* was reduced in KS Sertoli cells, but *NCL* remained unchanged (Figure 3I). The downregulation of *LRP1* was verified by qRT-PCR analysis of control and KS Sertoli cells (Figure 3J). The reduced MK pathway signaling from Leydig cells to Sertoli cells indicates possible important roles of the Leydig cell defects in KS testis.

### Delayed development of Leydig cells in KS fetal and adult testis.

**Figure 4:**
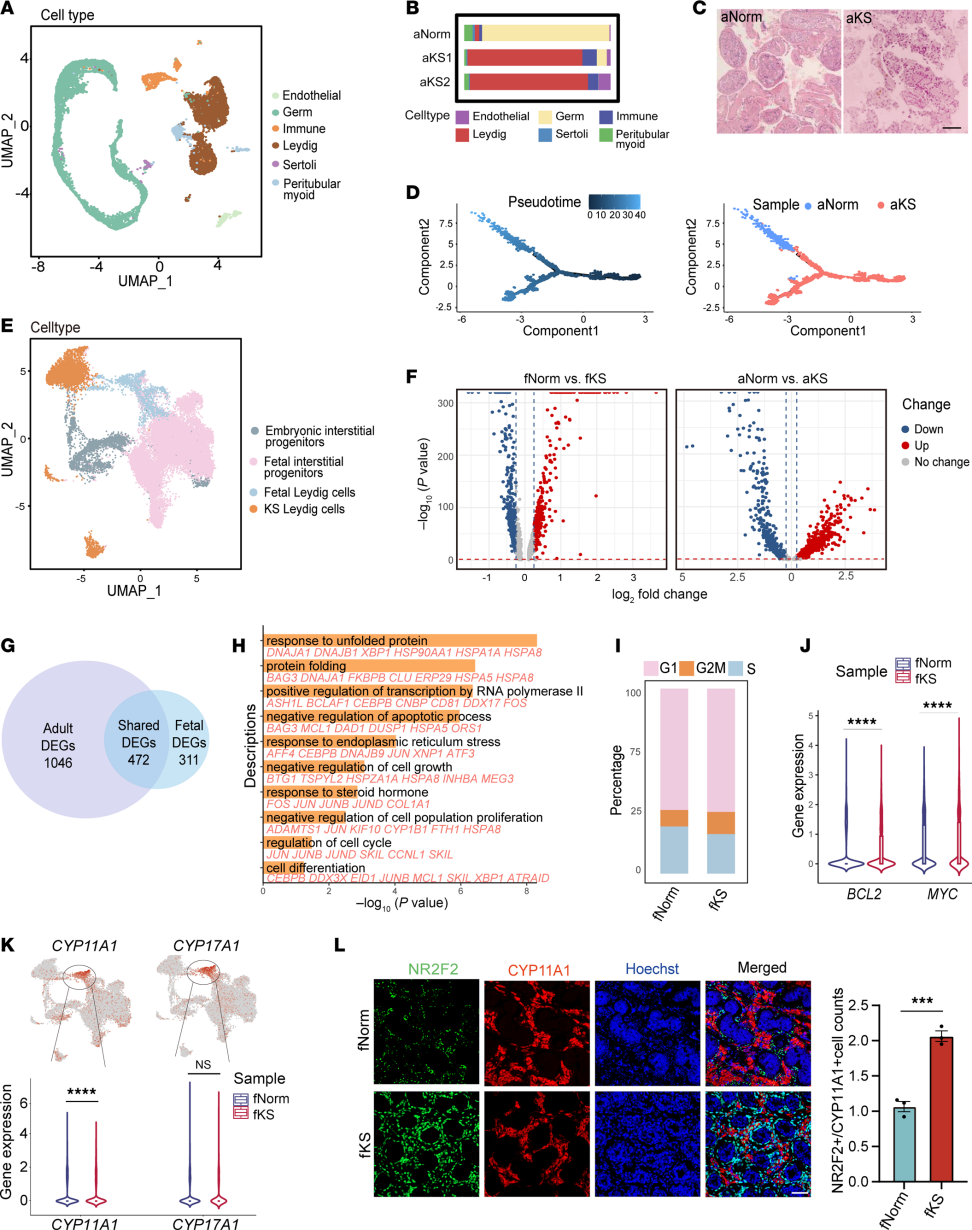
Delayed development of fetal Leydig cells in patients with KS. (**A**) UMAP plot of all cells in adults (3 samples). Dots represent individual cells, and colors represent different cell populations. (**B**) Cell ratio of cell types from **A**. (**C**) H&E staining of testicular biopsy samples from adult control (left) and KS (right) patients. (**D**) Pseudotime analysis of Leydig cells in adults. (**E**) UMAP plot of all Leydig cells in fetal stages (4 samples). Dots represent individual cells, and colors represent different cell populations. (**F**) Volcano plots compare control and KS Leydig cells for DEGs in fetuses (left) and adults (right). Genes with log_2_ (fold-change) above 0.25 or below –0.25 with *P* value lower than 0.05 were considered as having significantly differential expression. (**G**) Venn diagram of the 472 shared DEGs identified in both adult and fetal stages. (**H**) Bar plot showing GO terms enriched in shared DEGs. (**I**) Cell ratio of cells in the G1, S, and G2/M phases. (**J**) The expression level of *MYC* and *BCL2* with significant difference between control and KS samples. Statistical analysis was performed using 2-sided Wilcoxon’s rank-sum test; ****P < 0.0001. (**K**) The expression level of *CYP17A1* and *CYP11A1* with significant difference between control and KS samples. Statistical analysis was performed using 2-sided Wilcoxon’s rank-sum test; ****P < 0.0001. (**L**) Left: Immunofluorescence costaining of NR2F2 (green) and CYP11A1 (red). Scale bars, 50 μm. Right: Data are presented as the mean ± SEM from 3 independent experiments. ***P < 0.001 (*n* = 3).

Previous studies have reported that adult KS testis exhibits a reduction or even absence of germ cells, along with hyperplasia of Leydig cells and impaired androgen secretory function (35). To further explore these findings, we performed scRNA-seq analysis of adult KS testes, and we annotated 6 major cell types, including Leydig cells (*DLK1*), Sertoli cells (*SOX9*), germ cells (*DAZL*), endothelial cells (*VWF*), immune cells (*TYROBP*), and peritubular myoid cells (*RGS5*) (Figure 4A and Supplemental Figure 3, A and B). The proportions of cell types showed reduction in germ cells and increase in Leydig cells in adult KS testis (Figure 4B). H&E staining revealed a substantial loss of seminiferous tubules in adult KS testis, with germ cell depletion and extensive interstitial hyperplasia (Figure 4C).

Pseudotime analysis of scRNA-seq of adult KS testis demonstrated that KS Leydig cells appeared at the beginning of the developmental trajectory, whereas control Leydig cells were located mainly at the end (Figure 4D). Meanwhile, compared with the control group, adult KS testis contained 4 additional clusters of Leydig cells, which coincidentally also existed at the fetal stage (Supplemental Figure 3C). All of the above suggest that Leydig cells in adult patients with KS exhibit immature developmental state. We further analyzed the molecular markers of Leydig cells during the fetal period and identified 4 subpopulations, including embryonic Leydig progenitor cells (*MKI67*), fetal Leydig progenitor cells (*DLK1*), fetal Leydig cells (*CYP17A1*), and KS Leydig cells (Figure 4E and Supplemental Figure 3, D and E). We examined 472 shared DEGs in both adult and fetal stages (Figure 4, F and G, and Supplemental Tables 4 and 5), which were enriched in biological processes of cell cycle regulation, apoptosis, and response to steroid hormone (Figure 4H and Supplemental Table 6). This finding suggests that the impairment of Leydig cells in KS may originate from fetal development, with abnormal gene expression patterns established early and potentially exacerbated in adulthood.

One of the major manifestations of KS is the massive proliferation of Leydig cells and even Leydig cell tumors in some patients in adulthood (36). We performed cell cycle analysis, and we found that KS Leydig cells exhibited cell cycle changes, with an increase in G2/M-phase cells (Figure 4I). This indicated that the cells rapidly enter the division phase. We also observed abnormally elevated expression of *MYC* and *BCL2* genes in KS Leydig cells (Figure 4J and Supplemental Figure 3F). Previous studies showed that *MYC* and *BCL2* were abnormally expressed and led to abnormal cell proliferation in many cancers, suggesting that these 2 genes may play an important role in the phenomenon of carcinogenesis of Leydig cells observed in patients with KS (37, 38).

**Figure 5:**
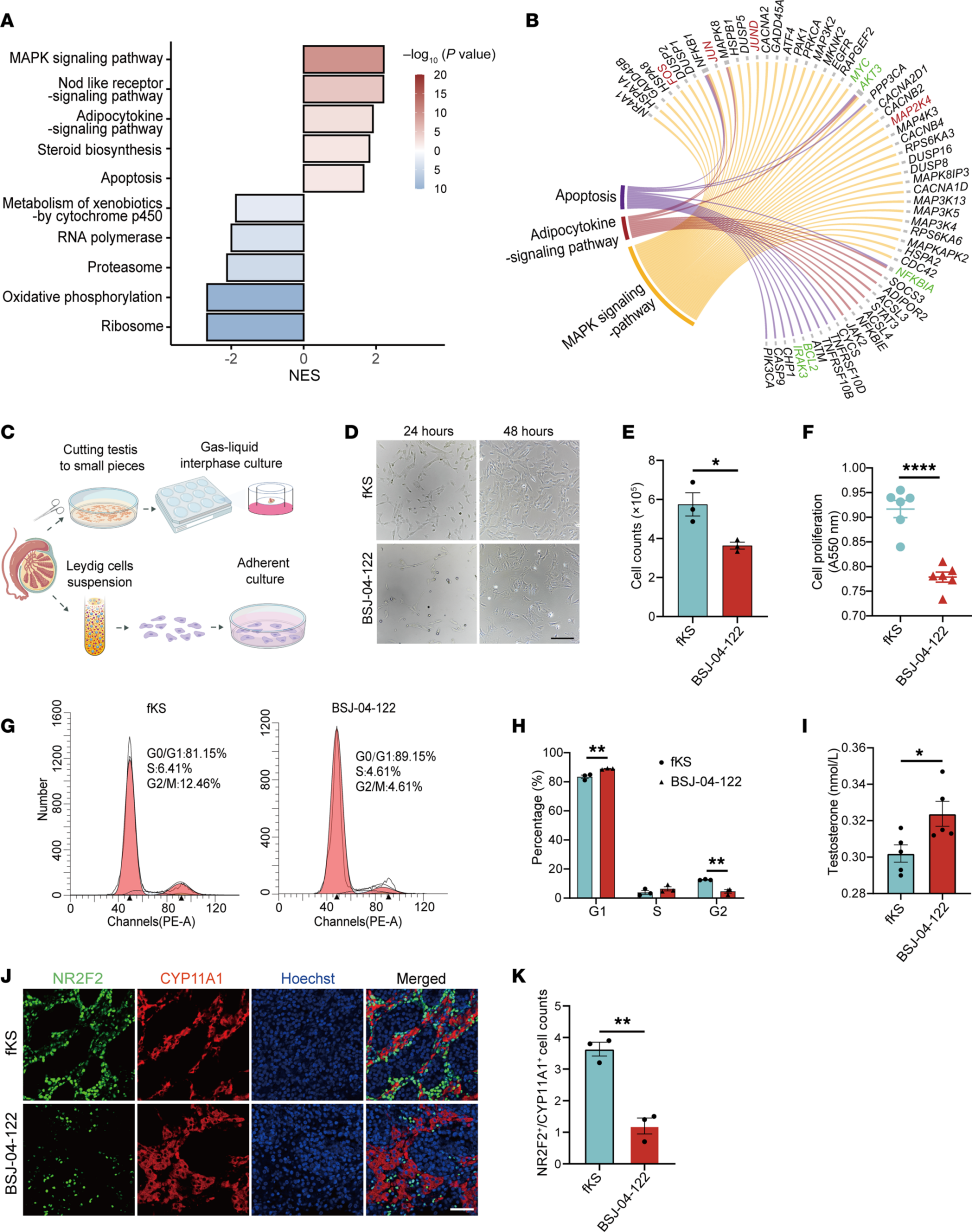
Inhibition of the MAPK pathway prevents overproliferation of KS Leydig cells and promotes testosterone production. (**A**) Bar plot showing the upregulated and downregulated Kyoto Encyclopedia of Genes and Genomes (KEGG) terms enriched in Leydig cells in the KS group compared with control. The length of the bar represents normalized enrichment score (NES). The color represents *P* value. (**B**) Chord diagram displaying the MAPK pathway and its associated genes. (**C**) Schematic diagram of fetal testis as well as primary Leydig cell culture. (**D**) Light micrographs of KS primary Leydig cells cultured with BSJ-04-122 or DMSO for 24 and 48 hours. Scale bar: 50 μm. (**E**) Cell count of **D**. Data are expressed as the mean ± SEM from triplicate experiments. *P < 0.05 (*n* = 3). (**F**) Cell proliferation analysis of KS primary Leydig cells cultured with BSJ-04-122 or DMSO for 48 hours. Data are expressed as the mean ± SEM from 5 experiments. ****P < 0.0001. (**G**) Cell cycle analysis of KS primary Leydig cells cultured with BSJ-04-122 or DMSO for 48 hours by flow cytometry (*n* = 6). (**H**) Statistical data on the cell cycle of 2 groups. Data are presented as the mean ± SEM from 3 independent experiments. **P < 0.01 (*n* = 3). (**I**) Testosterone content in the supernatant of KS primary Leydig cells cultured with BSJ-04-122 or DMSO for 48 hours. Data are expressed as the mean ± SEM from 5 experiments. *P < 0.05 (*n* = 5). (**J**) Immunofluorescence costaining of NR2F2 (green) and CYP11A1 (red). Scale bars, 50 μm. (**K**) Statistical data of **J**. Data are presented as the mean ± SEM from 3 independent experiments. **P < 0.01.

Of particular note, another major phenotype of KS testis is impaired testosterone synthesis. CYP17A1 and CYP11A1 are key enzymes in the steroid hormone synthesis pathway (39), and they regulate the production of testosterone. The expression of *CYP11A1* was significantly reduced in KS Leydig cells (*P* < 0.05), but *CYP17A1* showed no difference (*P* > 0.05) (Figure 4K). We further analyzed fetal Leydig progenitor cells and fetal Leydig cells in KS testes by IF staining, using NR2F2 to mark progenitors and CYP11A1 to mark fetal Leydig cells. The results showed increased percentage of fetal Leydig progenitor cells and decreased percentage of fetal Leydig cells in KS testis (Figure 4L). The gene set enrichment analysis (GSEA) further indicated enrichment of adipocytokine signaling pathways and steroid hormone synthesis pathways in KS Leydig cells (Figure 5A and Supplemental Table 7). This finding suggests disturbances of fat metabolism and hormone regulation in Leydig cells. Taken together, these results support abnormal cell proliferation and differentiation in KS Leydig cells, characterized by impaired maturation and hormone synthesis pathway defects.

### MAPK inhibition promotes the differentiation of KS Leydig cells.

To further investigate the proliferation and differentiation capacity of KS Leydig cells, we performed GSEA and found that the MAPK pathway was significantly upregulated in the KS group, including genes such as *MKK4*, *JUN*, and *FOS* (Figure 5, A and B, and Supplemental Figure 4, A and B).

KS Leydig cells exhibited defects in proliferation and testosterone secretion. To explore the roles of the MAPK pathway on the proliferation and differentiation of KS Leydig cells, we first isolated Leydig cells from KS fetal testis (Figure 5C). Morphological analysis and NR2F2 staining showed the purity of the isolated cells exceeded 86% (Supplemental Figure 4C). With KS Leydig cells, we used the inhibitor BSJ-04-122 to inhibit the MAPK pathway, and we evaluated cell number changes after 24 and 48 hours (Figure 5, D and E). The results showed that compared with the control group, the BSJ-04-122–treated group exhibited a significant reduction in cell numbers, indicating important roles of MAPK pathway in Leydig cell proliferation. The cell counting kit-8 (CCK-8) assay was also used to assess the proliferative activity, and the results demonstrated consistently reduced proliferative activity in the BSJ-04-122–treated group (Figure 5F). Additionally, we analyzed the cell cycle distribution of KS Leydig cells in the BSJ-04-122–treated and control groups using flow cytometry. The results revealed a significant decrease in the proportion of G2/M cells in the BSJ-04-122–treated group compared with the control group (Figure 5, G and H). The G2/M stage involves completion of DNA replication and preparation for mitosis. The reduction in G2/M cells suggests that the MAPK pathway inhibition interfered with the cell cycle progression, thereby inhibiting Leydig cell proliferation.

Fetal Leydig cells are major hormone-synthesizing cells in the fetal testis and play a key role in male sex development and reproductive system formation. We collected the culture medium of primary KS Leydig cells with and without BSJ-04-122 treatment, and we measured the levels of testosterone. We found that the concentration of medium testosterone increased significantly in the BSJ-04-122 treatment group, and inhibition of the upregulated MAPK pathway rescued the testosterone secretion disorder in KS Leydig cells (Figure 5I). To assess the testosterone synthesis capacity of KS Leydig cells, we performed qRT-PCR for *CYP11A1*, *CYP17A1*, *STAR*, and *3B-HSD*. Results revealed a significant upregulation in the BSJ-04-122–treated group for all targets except *CYP17A1* (Supplemental Figure 4D). The upregulated MAPK pathway impaired proliferation and differentiation of KS Leydig cells. Meanwhile, we examined the effects of MAPK inhibitors in normal Leydig cells and reached the same conclusion: adding inhibitors reduced cell proliferation and increased testosterone secretion. This indicates that Leydig cell progenitor fate is balanced between proliferation and differentiation (Supplemental Figure 4, E–G).

To study ex vivo functions of the MAPK pathway, we utilized a gonad tissue culture system (Figure 5C) and treated KS fetal testis tissue with the inhibitor BSJ-04-122. After 7 days of culture, the ratio of NR2F2-positive fetal Leydig progenitor cells to CYP11A1-positive fetal Leydig cells in KS fetal testis significantly decreased (Figure 5, J and K). These results collectively support that upregulation of the MAPK pathway is an important cause of differentiation impairment of KS Leydig cells.

### X-linked EIF1AX promotes proliferation of Leydig cell line.

**Figure 6:**
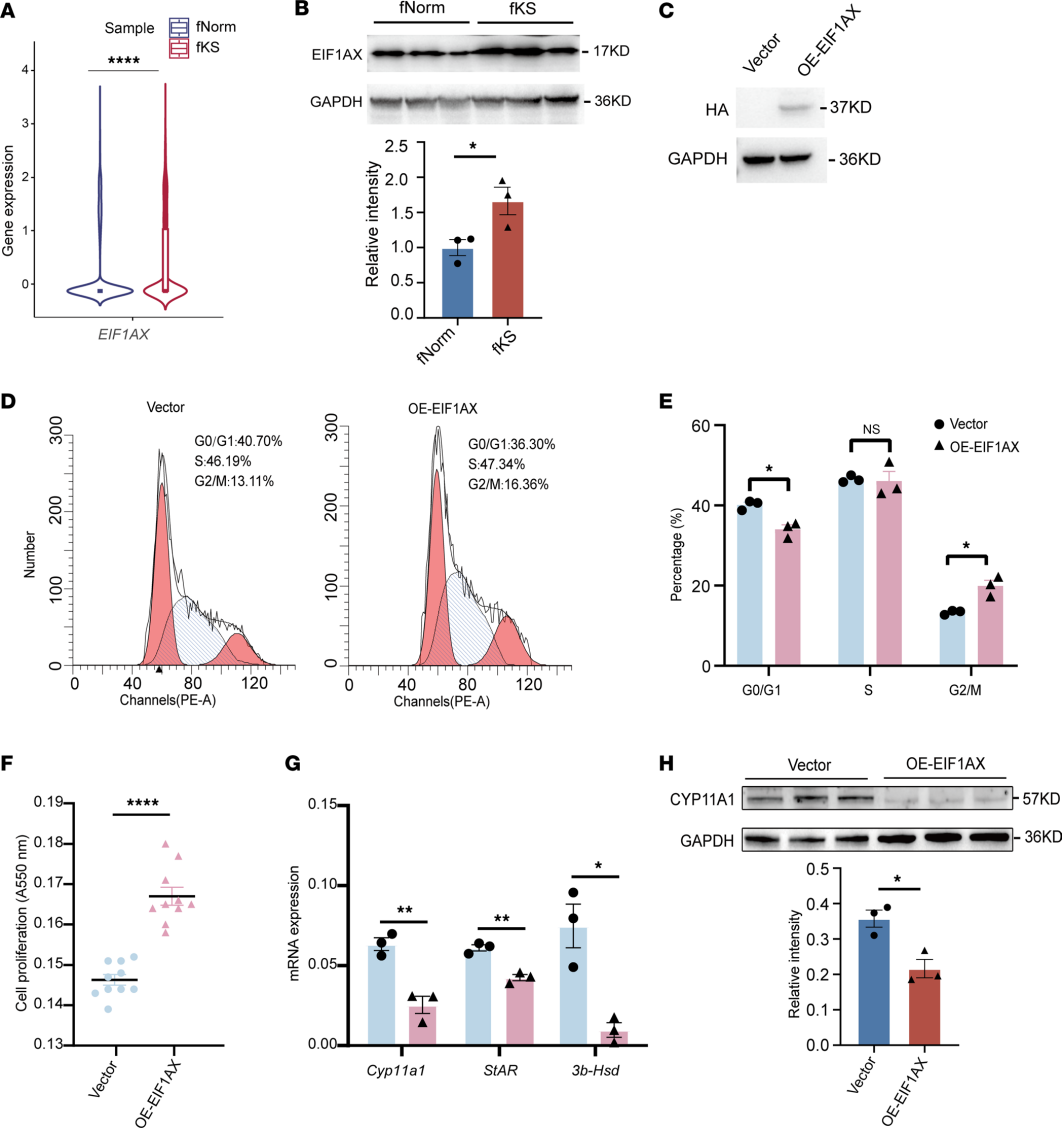
EIF1AX overexpression promotes TM3 cell proliferation and suppresses testosterone synthesis capacity. (**A**) The expression level of *EIF1AX* with significant difference between control and KS samples. Statistical analysis was performed using 2-sided Wilcoxon’s rank-sum test; ****P < 0.0001. (**B**) Western blot analysis of EIF1AX protein in KS fetal testis and control. Data are presented as the mean ± SEM from 3 independent experiments. *P < 0.05 (*n* = 3). (**C**) Western blot analysis of HA protein in control and EIF1AX-overexpressing TM3 cells. (**D**) Cell cycle analysis of control and EIF1AX-overexpressing TM3 cells by flow cytometry. (**E**) Statistical data on the cell cycle of 2 groups. Data are presented as the mean ± SEM from 3 independent experiments. *P < 0.05 (*n* = 3). (**F**) Cell proliferation analysis of control and EIF1AX-overexpressing TM3 cells. Data are expressed as the mean ± SEM from 10 experiments. ****P < 0.0001. (**G**) The expression of *Cyp11a1*, *StAR*, and *3b-Hsd* in control and EIF1AX-overexpressing TM3 cells using qRT-PCR. Data are presented as the mean ± SEM from 3 independent experiments. *P < 0.05. **P < 0.01. *GAPDH* was used as a loading control (*n* = 3). (**H**) Western blot analysis of CYP11A1 protein in control and EIF1AX-overexpressing TM3 cells. Data are presented as the mean ± SEM from 3 independent experiments. *P < 0.05 (*n* = 3).

Among 472 DEGs between KS and control Leydig cells in both adult and fetal stages (Figure 4G), we found that *EIF1AX*, a gene located on the X chromosome, was abnormally elevated in KS Leydig cells (Figure 6A). To evaluate the expression changes of EIF1AX at the protein level, we performed Western blot analysis. The results showed that EIF1AX was higher in fetal KS Leydig cells than in normal Leydig cells at protein levels (Figure 6B). Previous studies have reported upregulation of *EIF1AX* in KS blood samples (14). Furthermore, EIF1AX overexpression was reported to cause cell cycle dysregulation and increase cell proliferation in breast cancer (40). To further explore the function of EIF1AX in Leydig cells, we measured proliferation and hormone synthesis capacity in *Eif1ax*-overexpressing TM3 cells (Figure 6C and Supplemental Figure 4H). We found that EIF1AX overexpression upregulated the cell proliferation based on the CCK-8 assay (Figure 6F). Flow cytometry analysis showed that *Eif1ax* overexpression increased the transition from G0/G1 to S+G2/M phases, especially G2/M phase (Figure 6, D and E). To test the testosterone synthesis ability of TM3 cells, we performed qRT-PCR for *Cyp11a1*, *StAR*, and *3b-Hsd*, and we found a general decrease in gene expression (Figure 6G). Subsequently, we performed Western blot assay for CYP11A1 and found that *Eif1ax* overexpression resulted in a decrease in its protein expression in TM3 cells (Figure 6H). With an extra X chromosome in KS Leydig cells, upregulation of X-linked EIF1AX may be responsible for the aberrant proliferation of Leydig cells by facilitating the transition from G0/G1 to S+G2/M phases, which affects their hormone synthesis capacity.

## Discussion

Our scRNA-seq analysis of KS testis reveals Sertoli cell dysfunction and abnormal proliferation and differentiation of Leydig cells at the fetal stage. High level of MAPK signaling pathway and X-linked EIF1AX result in delayed Leydig cell development and decline in testosterone synthesis capacity. Inhibition of the MAPK activation pathway suppresses the overproliferation of KS Leydig cells and partially rescues testosterone production. The disorders of testicular microenvironment emerge during the fetal gonadal development.

Based on single-cell transcriptomics, we found that the number of prespermatogonia in patients with KS was reduced during the fetal period, consistent with previous research (18). In addition, we found that the upregulation of X chromosome–related genes may be an important factor contributing to the reduction of prespermatogonia. For example, the X-linked gene *HUWE1*, an E3 ubiquitin ligase, may induce apoptosis in primordial germ cells through P53 or caspase-3 (41, 42). Similarly, *ATRX*, another X-linked gene, has been reported to regulate germ cell apoptosis via caspase-3 (43).

We found 2 types of Sertoli cells in fetal KS testis: one type was negative for *XIST* with cell cycle dysregulation, whereas the other type was positive for *XIST* but with cell cycle distribution similar to normal fetal Sertoli cells. The existence of an extra X chromosome can cause overexpression of X-linked genes that escape inactivation, leading to pathological cascades (44). Our data showed resemblance of the *XIST-*positive KS Sertoli cell population to normal Sertoli cells, and both expressed low levels of *FOSB*. However, *XIST-*negative KS Sertoli cells showed a distinct cluster different from normal Sertoli cells. It has been documented that a subset of Sertoli cells in the testes of patients with KS remain developmentally immature (10). Typically, 2 distinct Sertoli cell populations are observed: those that are *XIST*-negative and those that are *XIST*-positive. Moreover, the expression of XIST continues to decline with advancing age (11–13). Mahyari et al.’s single-cell analysis of adult KS testis found that *XIST*^+^*SOX9*^+^ cells tended to exist in normal seminiferous tubules, while *XIST*^–^*SOX9*^+^ cells tended to reside in thick-walled seminiferous tubules with few spermatogenic cells (30). Our data supported the existence of both *XIST*-positive and *XIST*-negative Sertoli cell populations in KS testis and indicated Sertoli cell heterogeneity in X chromosome inactivation status begins at the fetal stage. *XIST*-negative Sertoli cell subpopulation exhibited aberrant X-linked gene upregulation, and abnormal overexpression of *FOSB*, a component of the AP-1 complex. The AP-1 complex can regulate cell proliferation (45–48), and its defect might contribute to the observed cell cycle abnormality of *XIST*-negative Sertoli cells.

Our scRNA-seq data revealed abnormally increased number of Leydig cells in both fetal and adult KS testes. In the adult KS testis, our pseudotime analysis of scRNA-seq data showed that Leydig cells remain developmentally immature. Previous studies of adult KS testis showed abnormal proliferation of Leydig cells (49, 50) and observed immature Leydig cells, abnormally differentiated Leydig cells, and multivacuolated Leydig cells (36). The immature Leydig cells in adult testis failed to produce testosterone (51). It seems that an abnormal number of KS Leydig cells are present from the fetal stage. We observed overlap of differentially expressed genes between fetal and adult KS Leydig cells, suggesting share abnormalities between the fetal and adult KS Leydig cells at the mRNA level. At the fetal stage, there were 3 populations of Leydig cells, including embryonic Leydig progenitor cells, fetal Leydig progenitor cells, and fetal Leydig cells (52). We found a significantly increased ratio of fetal Leydig progenitor cells but a decreased ratio of mature fetal Leydig cells in fetal KS testes. Patients with KS exhibit persistent Leydig cell abnormalities from fetal to adult stage, characterized by increased cell numbers, arrested development, and functional impairment.

Notably, we found marked upregulation of the MAPK pathway in KS Leydig cells. The MAPK pathway is known to regulate a variety of cellular processes, including proliferation, differentiation, apoptosis, and stress responses. It has been linked to impaired male fertility in response to harmful stimuli (53, 54). Odeh et al. demonstrated that activation of the MAPK/ERK cascade by PDGF-BB stimulates the proliferation of Leydig progenitor cells while inhibiting androgen production. In contrast, inhibition of the MAPK/ERK cascade by PDGF-AA promotes differentiation and steroidogenesis (55), indicating signaling pathways that drive mitotic proliferation typically suppress the expression of steroidogenic enzymes, and vice versa. Therefore, we utilized MAPK pathway inhibitors to inhibit the MAPK pathway, and the results showed that it ameliorates various phenotypes in both fetal KS and normal Leydig cells. It provides a potential regulatory target to improve the microenvironment and help develop the treatment of the disease.

In addition, we found that the X-linked gene *EIF1AX* shows aberrant overexpression in fetal KS Leydig cells, a finding confirmed at the protein level by Western blot analysis. The finding is consistent with the role of the EIF1A family in the regulation of cell proliferation. In drosophila, studies have shown that high expression of eIF1A in hopTum-l mutant larvae leads to hematopoietic dysfunction, differentiation abnormalities, and defective melanin deposition (56). In addition, in breast cancer, *EIF1AX* transcriptionally represses p21 through a p53-independent pathway, thereby driving tumor cell proliferation (40). Our work showed Leydig cells in KS testis are hyperproliferative with significant *EIF1AX* upregulation, suggesting its functional impact on Leydig cell proliferation/differentiation. In TM3 Leydig cells, we demonstrated that *Eif1ax* overexpression increased cell proliferation while reducing testosterone synthesis. Although the TM3 cell line is a mouse Leydig cell line derived from mouse immature Leydig tumors, which may not fully recapitulate the physiology of human fetal Leydig cells, the phenotype of *EIF1AX* overexpression recapitulates the abnormal phenotype of human KS Leydig cells. Previous studies reported that *EIF1AX* mutations could synergize with MAPK activation to enhance cell proliferation and tumorigenicity in low-grade tumors (57, 58). We found that similar to *EIF1AX* overexpression, activation of the MAPK pathway also increased cell proliferation and reduced testosterone synthesis. They may also synergize to cause the abnormal Leydig cell function in KS, and the detailed molecular mechanisms require further investigation.

Our scRNA-seq analysis of fetal testes was performed on only 2 KS samples because of the difficulty in obtaining samples. The low number of samples limits the ability to assess the inherent phenotypic heterogeneity within patients with KS. Our findings mainly focus on the common changes among KS samples. For example, the observations of MAPK pathway activation and altered EIF1AX expression in Leydig cells are consistent with the published data (18) and are also validated by additional independent KS samples through in vitro cell culture and/or tissue culture experiments. Future scRNA-seq analysis of a larger number of KS samples is expected to help us elucidate the heterogeneity of KS.

In summary, our scRNA-seq analysis of human KS testis showed abnormal proliferation/differentiation of KS Leydig cells, and inhibition of the overactivated MAPK signaling pathway or the X-linked gene *EIF1AX* could help reduce the phenotypes. These findings showed abnormalities of microenvironment for germ cell development started from the fetal stage. Our study provides a theoretical basis for the molecular mechanisms of KS-associated infertility and lays an important scientific foundation for the development of early intervention strategies against this disease.

## Methods

### Sex as a biological variable.

All experiments were performed using male samples. Because this study focuses on testicular developmental abnormalities in KS, all KS and control samples are male. Sex was not considered as a biological variable in this study.

### Sample preparation for scRNA-seq.

The fetal testes were identified near the internal inguinal ring. The fetal testis tissues were handled within 24 hours of abortion. The diagnosis of sex was based on the morphology of the internal and external genitalia. The age of the fetus was based on the gestational time calculated by the time of the last menstrual period and the embryo size measured by the ultrasound. The KS fetuses were diagnosed by karyotyping of amniotic fluid cells using the chromosome copy number variation detection kit (TIANGEN) (Supplemental Figure 1A). Trisomy 21, trisomy 18, monosomy X, 47,XYY syndrome, or other chromosomal abnormalities were not included in this study. Among the 15 fetal testis samples subjected to screening, 10 were used in this study. The testis tissues were incubated in 1 mL of accutase cell separation solution (Yeasen, 40506ES60) at 37°C for 10 minutes, terminated by the addition of 1 mL of DMEM/F12 containing 10% FBS, and purified through a 40 μm cell filter (Biosharp, BS-40-XBS). Cells were centrifuged at 500*g* for 5 minutes and resuspended in PBS twice. Finally, the cells were counted using an automatic cell counter (Thermo Fisher Scientific, Countess II). The cells were adjusted to 4 × 10^5^ cells/mL in concentration and used immediately for scRNA-seq.

### ScRNA-seq and raw data analysis.

This experiment was performed according to the user manual for the GEXSCOPE Single Cell RNA Library Kit for SD/HD (Singleron). Single cells were captured using a SCOPE-chip microfluidic chip, and millions of Barcode Beads with unique cell barcodes were added to the microtiter wells of the chip to ensure that only 1 barcode bead fell into each microtiter well. After the cells were lysed, barcode beads with unique cell barcodes and unique molecular identifiers (UMIs) were added to the microtiter wells of the chip. After cell lysis, barcode beads carrying unique cell barcodes and UMIs captured mRNAs by binding to their poly(A) tails, thereby labeling each mRNA with a cell specific barcode and a UMI. The barcode beads were collected from the microarray, and the mRNAs captured by the barcode beads were reverse-transcribed into cDNAs and amplified. The cDNAs were fragmented and ligated to construct a sequencing library for the Illumina sequencing platform.

The CeleScope (v.1.14.1) pipeline provided by Singleron was used to manipulate raw sequencing reads that were demultiplexed and aligned to the hg38 genome. A gene-cell UMI matrix was then generated for each sample.

### Histological analysis.

Tissues were fixed overnight in 4% paraformaldehyde at 4°C. The fixed tissues were processed through gradient dehydration and subsequently embedded in paraffin. Paraffin-embedded tissues were sectioned, and the tissue sections were placed on slides. H&E staining was performed and the tissue morphology was observed under a microscope (ZEISS Axio ImagerA1).

### IF staining.

Before staining, tissue sections were dewaxed in xylene, rehydrated using a gradient series of ethanol solutions, and washed in distilled water. After incubating the tissue surface with 3% BSA/PBS+0.1% Triton for 30 minutes at room temperature, we stained with the primary and secondary antibodies. Primary and secondary antibodies used in the study are provided in Supplemental Table 8. Nuclei were labeled with Hoechst by incubating tissue sections for 1 minute. Slides were visualized with an LSM700 confocal microscope (ZEISS).

### Western blot analysis.

Protein samples were prepared with RIPA lysis buffer (Beyotime, P0013C) with protease inhibitor cocktail (Bimake, B14001) and phosphatase inhibitor (Sigma, P5726). After preparation, the samples were subjected to electrophoresis and transferred onto a nitrocellulose membrane (Bio-Rad, 1620177). Subsequently, the membranes were blocked with 5% skimmed milk and incubated with corresponding primary antibodies and secondary antibodies. Protein bands were detected using an ECL kit (Thermo Fisher Scientific, 32109) and a Bio-Rad ChemiDoc XRS+ gel imaging system.

### Cell culture.

TM3 cells (ATCC, CRL-1714) were used in this study. Cells were grown in DMEM at 37°C with a 5% CO_2_ atmosphere. The medium was supplemented with 10% FBS, as well as penicillin at a concentration of 100 U/mL and streptomycin at 100 μg/mL. Transfection was accomplished using ExFect Transfection Reagent (Vazyme) according to the manufacturer’s instructions.

### Isolation and characterization of Leydig cells from fetal testes.

The isolation of Leydig cells from fetal testes was performed using an enzymatic digestion combined with adhesion method. Briefly, fresh fetal testicular tissue was stripped off the tunica albuginea under sterile conditions and minced into approximately 1 mm^3^ fragments. The tissue fragments were sequentially digested in F12 medium (Gibco) containing collagenase IV (1 mg/mL) (Thermo Fisher Scientific) and deoxyribonuclease I (20 μg/mL) (Thermo Fisher Scientific) at 37°C with shaking at 120 rpm for 30 minutes. An equal volume of medium was added to terminate the digestion. The cell suspension was filtered through a 40 μm strainer and centrifuged at 300 × *g* for 10 minutes. Subsequently, the cell suspension was cultured in DMEM/F12 medium supplemented with 10% FBS and 1% antibiotic-antimycotic (Gibco) in a 37°C incubator. IF staining was performed using specific antibodies against the fetal Leydig cell marker NR2F2. Primary and secondary antibodies used in the study are provided in Supplemental Table 8. Over 85% of the adherent cells were tested positive, confirming their identity as Leydig cells. On average, each fetal testicular sample yielded 0.8 × 10^5^–1.5 × 10^5^ positively identified Leydig cells following this procedure.

### Fetal gonad culture.

We added 400 μL of medium to the bottom chamber of each 24-well Transwell dish (3422, Corning) and incubated at 34°C for at least 1 hour with 5% CO_2_. Gonads from KS and male control fetuses were minced and rinsed 3 times in PBS solution. Culture medium (10% FBS, 0.46% sodium pyruvate, 0.05 mg/mL vitamin C dissolved in 10 mL of α-MEM medium) was added to a 24-well plate and inserted into the culture chambers, and the gonads were carefully placed into the chambers using forceps and incubated in a 34°C/5% CO_2_ incubator, with the culture medium changed every 2 days. For phenotypic rescue, the medium was supplemented with 10 μM BSJ-122-04 (MAPK pathway inhibitor) (MedChemExpress [MCE], HY-152185). For other tissue culture, an equal amount of 0.1% DMSO solvent was added to the medium.

### qRT-PCR.

qRT-PCR was performed using a SYBR Green Premix Pro Taq HS qPCR Kit (Accurate Biotechnology). The relative transcript levels of samples were compared with the controls. For each experiment, qRT-PCRs were carried out in triplicate. Relative expression level was determined with 2^–ΔΔCt^, using *GAPDH* as a loading control. Primers used in qRT-PCR are provided in Supplemental Table 9.

### Cell proliferation assay and cell cycle.

Cells were digested with trypsin and cultured in 96-well plates at a density of 1 × 10^4^ cells/well. Cell proliferation assay was performed using CCK-8 (Vazyme, A311-01). For cell cycle analysis, cultured cells were digested with trypsin, washed with cold PBS, and fixed with precooled 70% ethanol in PBS at 4°C for 24 hours. Cells were precipitated by centrifugation at 600 × *g* and resuspended in cold PBS twice. Last, the cells were incubated with 20 μg/mL propidium iodide (MCE, HY-K1071) for 30 minutes at 37°C, protected from light. The cell cycle distribution of each sample was analyzed using a FACSCalibur flow cytometer (BD Biosciences).

### Cell type identification and clustering analysis using Seurat program.

First, the Seurat package (v.4.3.0) was applied to analyze the scRNA-seq data. High-quality cells were defined as those expressing more than 600 genes and less than 20% mitochondrial gene expression. We normalized expression matrices by both the number of reads and the cell number using the NormalizeData function in the Seurat package. The FindVariableFeatures function was then used to identify the top 2,000 variable genes for subsequent principal component analysis. Then data scaling was performed with ScaleData. We clustered cells on each repetitive sequence, resulting in similar UMAP profiles. Unsupervised clustering based on the Harmony-corrected reduced dimension space was performed by FindNeighbors function (dims=1:20) and FindClusters function (resolution=0.5) to generate UMAP visualizations. The Leydig, Sertoli, and germ cells were selected and then reclustered. The same processing steps were repeated to obtain subclusters.

### DEGs and pathway enrichment analyses.

DEGs for each cell type were acquired between male control and KS samples using FindMarkers function of Seurat package (v.4.3.0) at *P* < 0.05. GO enrichment analysis was applied to all DEGs and marker genes using the clusterProfile package (v.4.2.2), and terms with *P* < 0.05 were considered significantly enriched. GSEA was performed using the gseKEGG function in the clusterProfile package.

### Cell trajectory analysis.

The analyses were carried out with the Monocle software package (version 2.22.0), adhering to a standard workflow and the default settings. Cell trajectories were generated by utilizing the marker genes identified by Seurat package. Subsequently, the trajectories were visualized using the plot_cell_trajectory function.

### Cell-cell communication analysis.

The CellChat software package (v1.6.1) was used to analyze interaction probabilities and compare cellular communication between the male control and KS datasets based on the average gene expression per cell within each cell group. The CellChatDB.human was confirmed to be the ligand-receptor interaction database, and then cell-cell communication analysis was performed with the default settings. We used the mergeCellChat function to merge the CellChat objects for each group, comparing the total number of interactions and the strength of the interactions. Differences in the number of interactions or differences in the strength of interactions between different cell populations were visualized using the netVisual_diffInteraction function. Finally, the interactions between the specified cells were visualized using the netVisual_bubble function.

### Statistics.

Data were presented as mean ± SEM. Comparisons between 2 groups were conducted using a 2-tailed Student’s *t* test. *P* < 0.05 is considered statistically significant. The number of independent biological replicates (*n*) for each experiment is specified in the corresponding figure legend.

### Study approval.

Written informed consents were obtained from all donors. Prenatal male gonads used for scRNA-seq were obtained from aborted fetuses with the approval of the Ethical Committee of the Women’s Hospital of Nanjing Medical University (Permission Number 2021KY042). Fresh testicular puncture tissue was obtained from donors undergoing microdissection testicular sperm extraction with approval of the Ethical Committee of The First Affiliated Hospital of Nanjing Medical University (Permission Number 2023-SZ-01). Details of the samples are given in Supplemental Table 1.

### Data availability.

All data are available in the main text, supplement, and Supporting Data Values file. The scRNA-seq datasets have been deposited in the Genome Sequence Archive (59) in National Genomics Data Center (60), China National Center for Bioinformation/Beijing Institute of Genomics, Chinese Academy of Sciences (GSA-Human: HRA012784), and is now publicly available. The public may access it via https://ngdc.cncb.ac.cn/gsa-human

## Author contributions

TY, GC, JZ, WJ, and NL investigated. GC, JZ, WJ, NL, CQ, XS, and QC curated data. TY, CQ, XS, QC, and XG designed the study. HZ, YL, XY, and YZ prepared samples for scRNA-seq. TY, H Zhang, and YZ carried out scRNA-seq experiments. JZ, SJ, and LJ collected clinical samples. GC and NL conducted the cellular experiments. TY, WJ, QL, QW and JW performed data analysis. TY, JZ, H Zhu, CQ, XS, QC, and XG reviewed and edited the manuscript.

## Conflict of interest

The authors have declared that no conflict of interest exists.

## Funding support

- The National Natural Science Foundation of China grants 82530053, 82221005, 82371606 (to XG).
- The National Natural Science Foundation of China grants 82371623, 82571851 (to YL).
- The National Natural Science Foundation of China grant 32300716 (to CS).
- The National Natural Science Foundation of China grant 82271636 (to H Zhu).
- The National Natural Science Foundation of China grant 62273175 (to XS).
- The Natural Science Foundation of Jiangsu Province grants No BK20251858 (to YL).
- The Key R&D projects of Jiangsu Province grants No. BE2022843 (to XS).
- Jiangsu Health Development Research Center grants JSHD202424 (to QC).

## Supplementary Material

Supplemental data

Unedited blot and gel images

Supplemental table 1

Supplemental table 2

Supplemental table 3

Supplemental table 4

Supplemental table 5

Supplemental table 6

Supplemental table 7

Supplemental table 8

Supplemental table 9

Supporting data values

## Version 1 06/09/2026

In-Press Preview

## Version 2 07/15/2026

Electronic publication
