# Supplementary material for: Single-cell analysis of fetal testis reveals dysfunction of human Leydig cells in Klinefelter syndrome
Source: J Clin Invest. 2026 Jun 9;136(14):e201124. doi: 10.1172/JCI201124 (PMC13367963; doi:10.1172/JCI201124)
Supplement: Supplemental data [file jci-136-201124-s192.pdf]

Supplementary Materials

Single-cell analysis of fetal testis reveals dysfunction of human Leydig cells in Klinefelter syndrome

Supplementary Figures

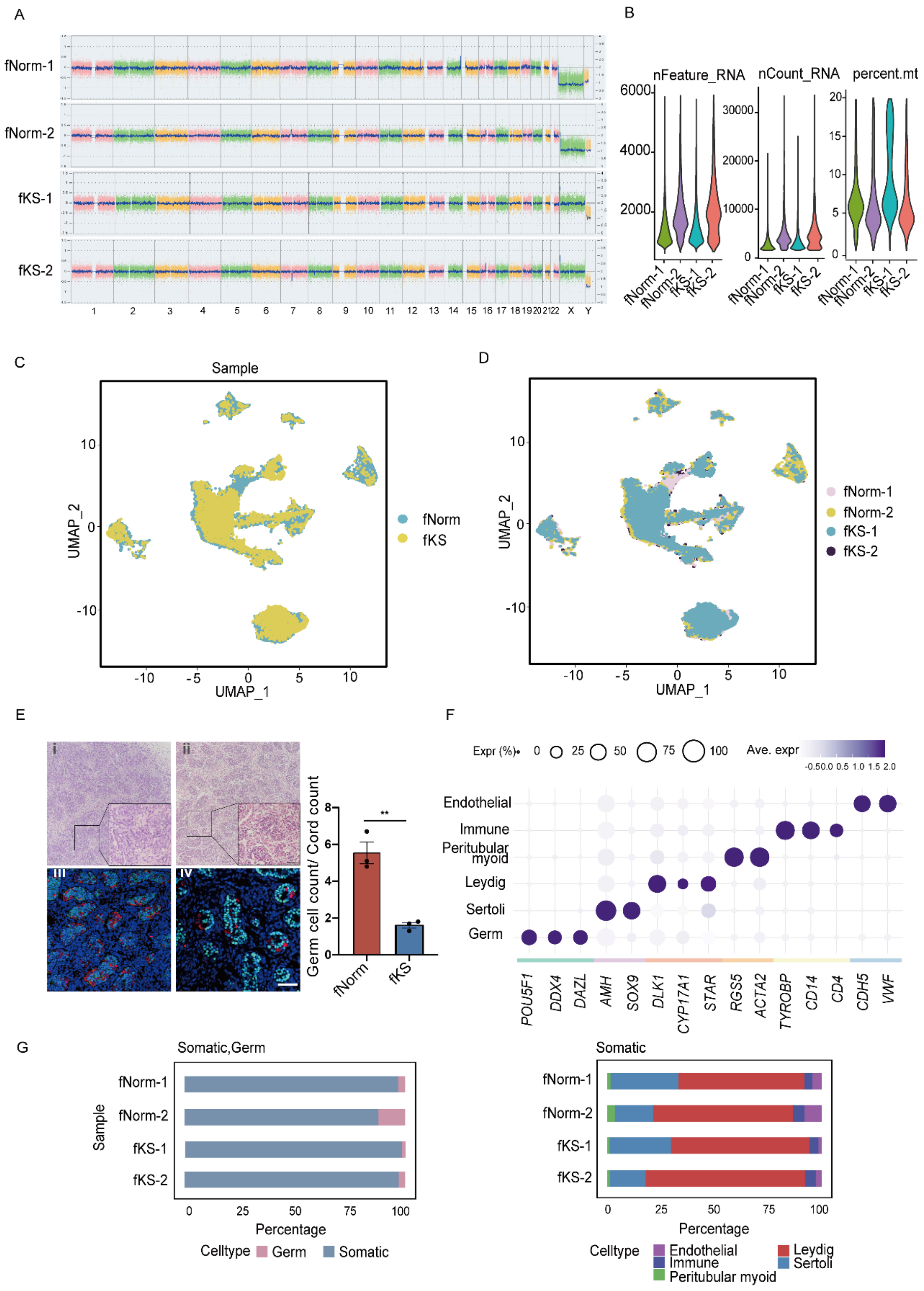

Figure S1. Quality control of single-cell RNA-seq datasets

(A) Copy number variation (CNV) analysis of the KS and normal fetuses determined by

karyotyping of amniotic fluid cells.

(B) Violin plots of global transcript levels for different samples.

(C) UMAP plot showing the major cell types from KS samples (n = 2) and samples from aborted male fetuses (n = 2). Each dot corresponds to an individual cell, and the colors denote different group.

(D) UMAP plot showing the major cell types from KS samples (n = 2) and samples from aborted male fetuses (n = 2). Each dot corresponds to an individual cell, and the colors denote different samples.

(E) HE staining of tissue sections from control (i) and KS groups (ii). IF staining of DDX4 and SOX9 in tissue sections of the control (iii) and KS groups (iv), revealing a significant reduction in the number of DDX4+ cells in the KS group. Scale bars, 50μm. The data are expressed as mean ± SEM based on 15 regions from three independent male control samples and 20 regions from three independent KS samples. Statistical analysis was performed using unpaired two-sided *t*-tests; **p < 0.01. Right:

(F) Dot plot showing the expression of selected gene markers for different cell cluster.

(G) Left: Cell ratio of somatic and germ cells. Right: Cell ratio of various somatic cells.

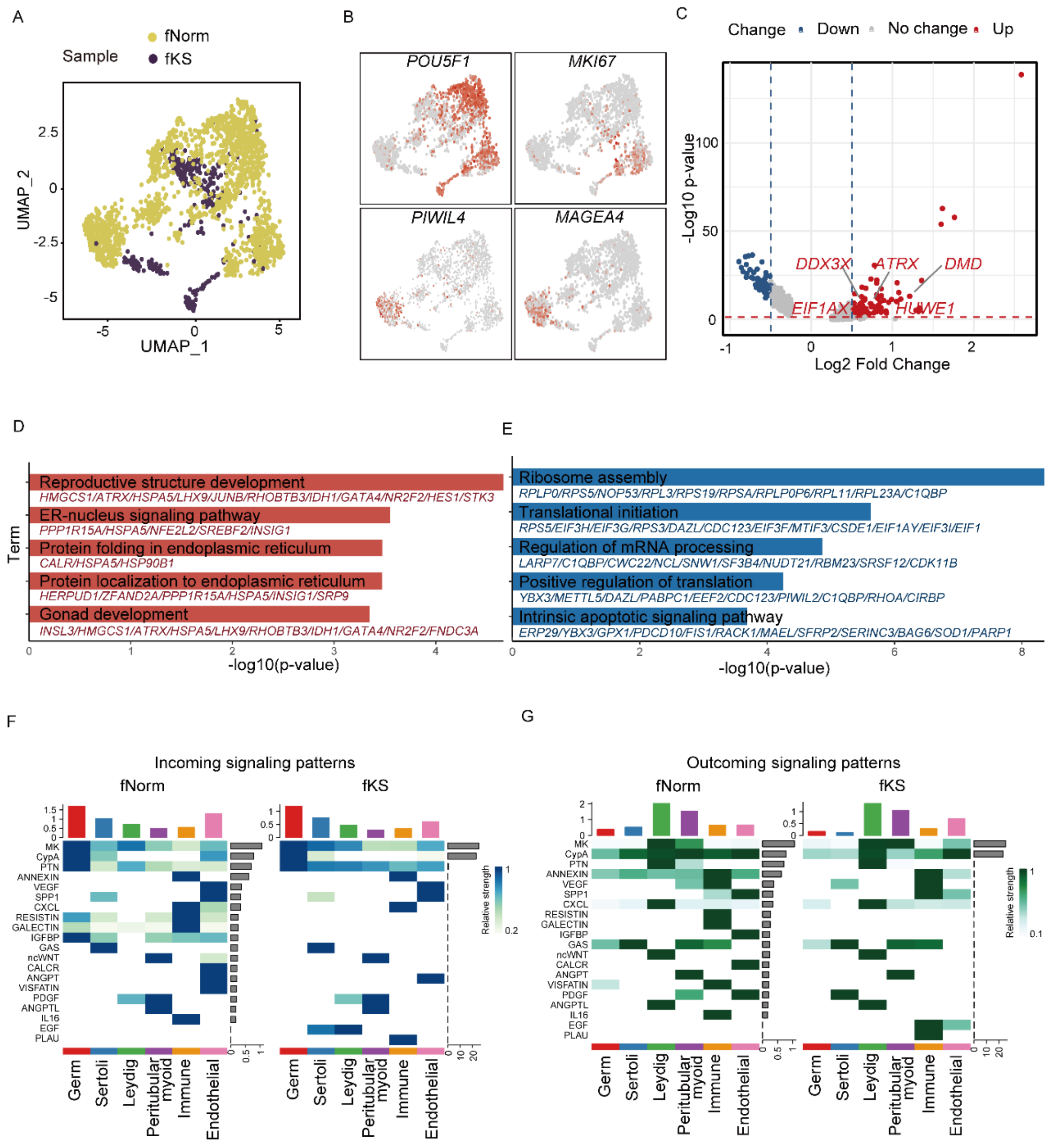

Figure S2. Germ cell dysfunction in KS

(A) UMAP plot of all germ cells from the fetal sample (4 samples). Each dot corresponds to an individual cell, and the colors denote different groups.

(B) Expression patterns of selected markers displayed on the UMAP plot.

(C) Volcano plots compare control and KS samples for differentially expressed genes in germ cells. Genes with log2 (fold-change) beyond 0.5 or below -0.5 with p-value lower than 0.05 were considered as significantly differential expression. Genes on the up-regulated X chromosome are labeled in red.

(D) Bar Diagram of Upregulated go term.

(E) Bar Diagram for Downregulated go term.

(F) The relative change in incoming signal pathway activity (KS vs control).

(G) The relative change in outcoming signal pathway activity (KS vs control).

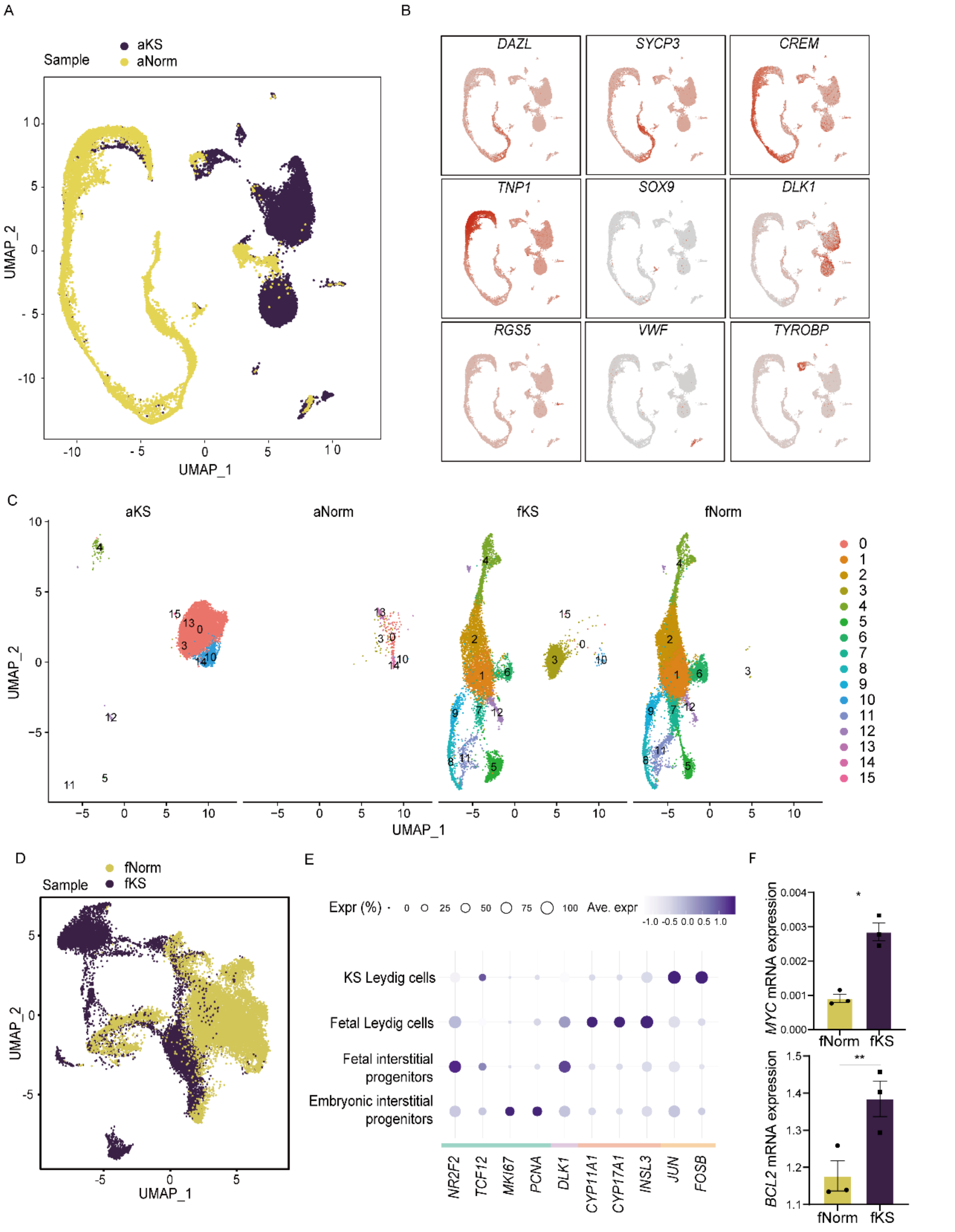

Figure S3. Leydig cells clusters of adult and fetal testes

(A) UMAP plot of all Leydig cells from the adult sample (3 samples). Each dot corresponds to an individual cell, and the colors denote different groups.

(B) Expression patterns of selected markers displayed on the UMAP plot.

(C) UMAP plots of all Leydig cells in each sample. Each dot corresponds to an individual cell, and the colors denote different clusters.

(D) UMAP plot of all Leydig cells from the fetal sample (4 samples). Each dot corresponds to an individual cell, and the colors denote different groups.

(E) Dot plot showing the expression of selected gene markers for different cell cluster.

(F) The expression level of *MYC* and *BCL2* with significant difference between control and KS Leydig cells. Statistical analysis was performed using unpaired two-sided *t*-tests; *p< 0.05, **p< 0.01. *GAPDH* was used as a loading control (n=3).

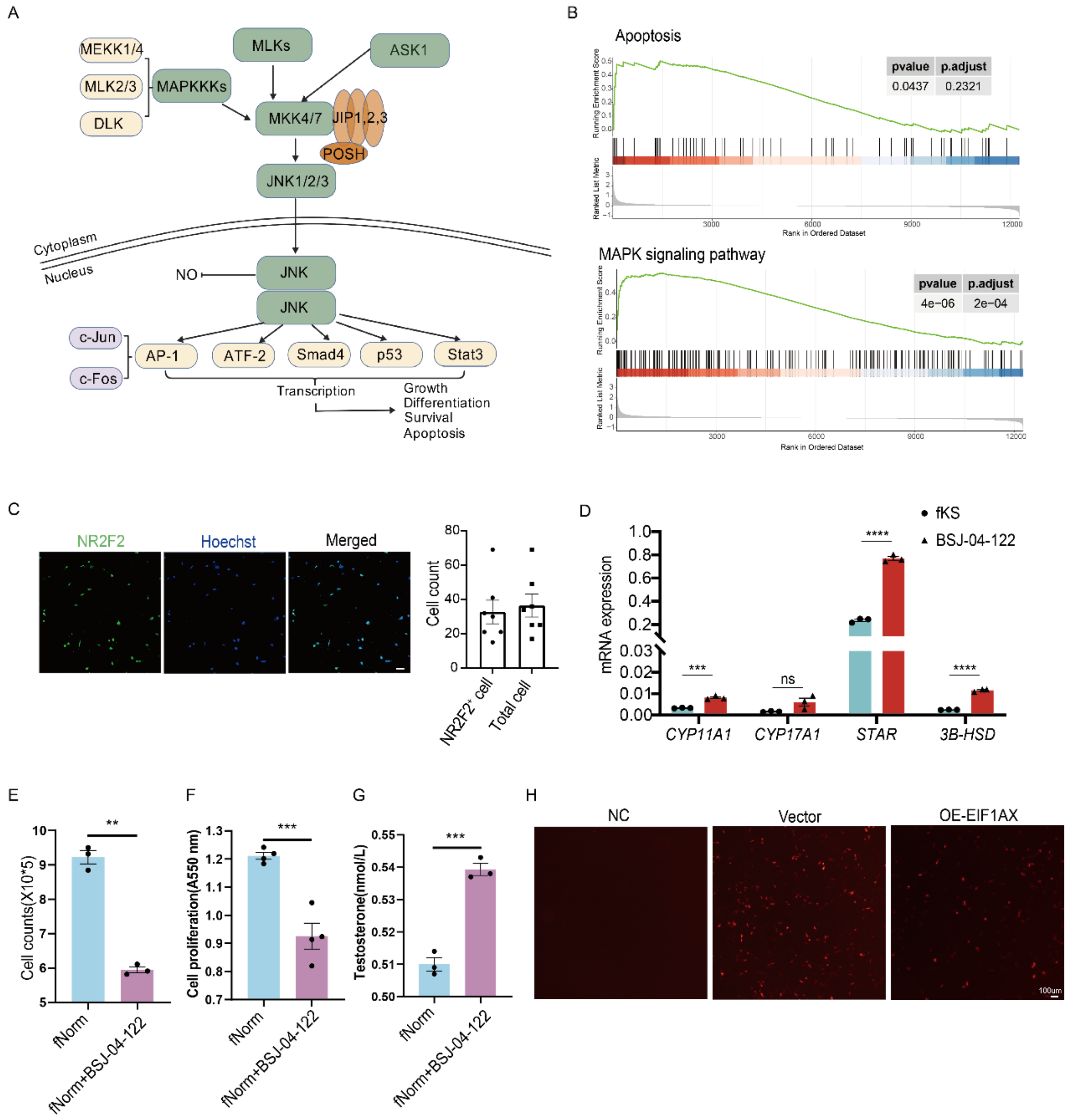

Figure S4. The role and mechanism of MAPK pathway and EIF1AX in KS Leydig cells

(A) Schematic diagram of MAPK pathway.

(B) GSEA score compare control and KS samples of Apoptosis and MAPK signaling pathway.

(C) IF staining of NR2F2 in tissue sections of primary Leydig cells isolated from KS fetal testis with cell counting. Scale bars, 50μm.

(D) The expression of *CYP11A1*, *CYP17A1*, *STAR* and *3B-HSD* in primary Leydig cells cultured with BSJ-04-122 or DMSO for 48 hours using qRT-PCR. Data are presented as the mean ± SEM from three independent experiments. ***p< 0.001. ****p< 0.0001. *GAPDH* was used as a loading control (n=3).

(E) Cell count of primary Leydig cells cultured with BSJ-04-122 or DMSO for 48 hours is expressed as the mean ± SEM from triplicate experiments. $**p < 0.01$ ($n=3$).

(F) Cell proliferation analysis of primary Leydig cells cultured with BSJ-04-122 or DMSO for 48 hours. Data are expressed as the mean ± SEM from quadruplicate experiments. $***p < 0.001$ ($n=4$).

(G) Testosterone content in the supernatant of primary Leydig cells cultured with BSJ-04-122 or DMSO for 48 hours. Data are expressed as the mean ± SEM from triplicate experiments. $***p < 0.001$ ($n=3$).

(H) Fluorescence expression in TM3 cells after electro-transfection. Scale bars, 50μm.

Supplementary Tables

Table S1. Detailed clinical samples

Table S2. GO annotation of marker genes

Table S3. GO annotation of three types of Sertoli cells in fetal KS and control testis

Table S4. Differentially expressed genes of KS Leydig cells in fetal stage

Table S5. Differentially expressed genes of KS Leydig cells in adult stage

Table S6. GO annotation of differentially expressed genes in KS Leydig cells shared in fetal and adult stages

Table S7. KEGG enrichment analysis of differentially expressed genes in fetal KS Leydig cells

Table S8. Antibodies used in this study

Table S9. Primers used in this study
