# Supplementary figures and images for: Single-cell analysis of fetal testis reveals dysfunction of human Leydig cells in Klinefelter syndrome
Source: J Clin Invest. 2026 Jun 9;136(14):e201124. doi: 10.1172/JCI201124 (PMC13367963; doi:10.1172/JCI201124)

Figure6B

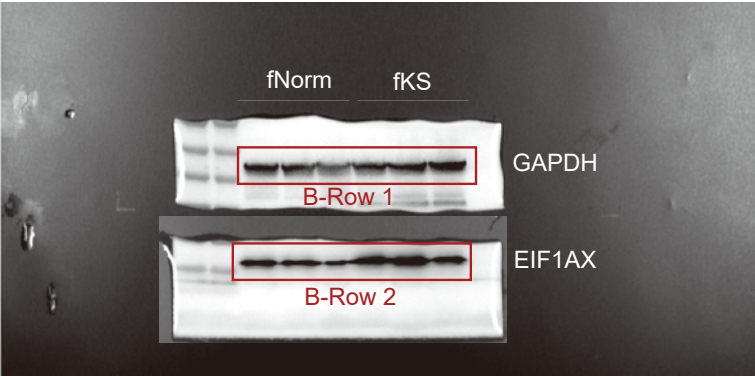

Figure6C

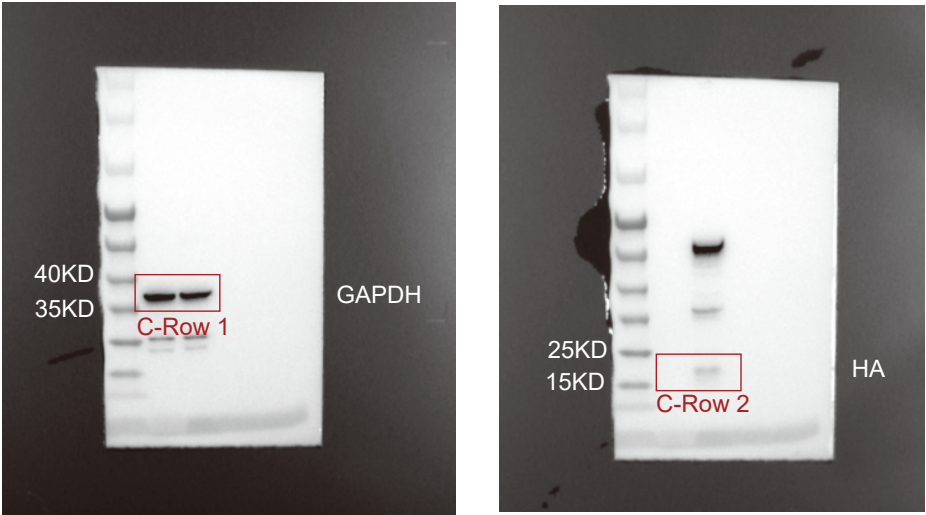

Figure6H

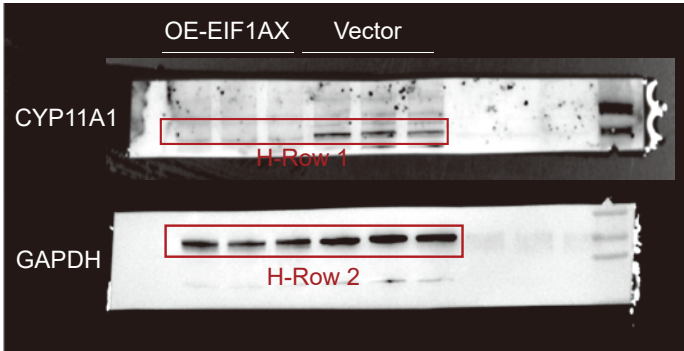

Supplement: Unedited blot and gel images [file jci-136-201124-s193.pdf]
